# BRASERO: A Resource for Benchmarking RNA Secondary Structure Comparison Algorithms

**DOI:** 10.1155/2012/893048

**Published:** 2012-05-23

**Authors:** Julien Allali, Cédric Saule, Cédric Chauve, Yves d'Aubenton-Carafa, Alain Denise, Christine Drevet, Pascal Ferraro, Daniel Gautheret, Claire Herrbach, Fabrice Leclerc, Antoine de Monte, Aida Ouangraoua, Marie-France Sagot, Michel Termier, Claude Thermes, Hélène Touzet

**Affiliations:** ^1^LaBRI, UMR 5800 CNRS, Université Bordeaux, 351, Cours de la Libération, 33405 Talence Cédex, France; ^2^The Pacific Institute for the Mathematical Sciences, University of British Columbia, CNRS UMI 3069, 200-1933 West Mall Vancouver, BC, Canada V6T 1Z2; ^3^LRI, UMR 8623 CNRS, Université Paris-Sud and INRIA Saclay, 91405 Orsay Cédex, France; ^4^Department of Mathematics, Simon Fraser University, 8888 University drive, Burnaby, BC, Canada V5A 1S6; ^5^Centre de Génétique Moléculaire, UPR 3404 CNRS, Avenue de la Terrasse, Bât. 26, 91198 Gif-Sur-Yvette, France; ^6^IGM, CNRS UMR 8621, Université Paris-Sud, 91405 Orsay Cédex, France; ^7^MAEM, CNRS UMR 7567, Université Henri Poincaré, 1 Boulevard des Aiguillettes, BP 239, 54506 Vandoeuvre-Les-Nancy Cédex, France; ^8^LIFL, CNRS UMR 8022, Université Lille 1 and INRIA, 59655 Lille Cédex, France; ^9^Inria Rhône-Alpes and LBBE, UMR 5558 CNRS, Université Claude Bernard, Bât. Grégor Mendel, 43 Boulevard du 11 Novembre 1918, 69622 Villeurbanne Cédex, France

## Abstract

The pairwise comparison of RNA secondary structures is a fundamental problem, with direct application in mining databases for annotating putative noncoding RNA candidates in newly sequenced genomes. An increasing number of software tools are available for comparing RNA secondary structures, based on different models (such as ordered trees or forests, arc annotated sequences, and multilevel trees) and computational principles (edit distance, alignment). We describe here the website BRASERO that offers tools for evaluating such software tools on real and synthetic datasets.

## 1. Introduction

Motivated by the fundamental role of RNAs, and especially of small noncoding RNAs, several methods for high-throughput generation of noncoding RNA candidates have been developed recently [1–3]. A fundamental problem is then to infer functional annotation for such putative RNA genes [4, 5] which often involves RNA structure comparisons. Most approaches to compare RNA structures focus on the secondary structure, an intermediate level between the sequence and the full three-dimensional structure, which is both tractable from a computational point of view and relevant from a functional genomics point of view. The problem we consider here is the following: given a new RNA secondary structure (*the query*) and a database of known and annotated RNA secondary structures which of these known structures display most structural features similar to the query? Databases such as RFAM [6] or RNA STRAND [7] come naturally to mind, but in-house collections of RNA structures resulting from high-throughput experiments can also be considered.

Fundamentally, mining a database of RNA secondary structures naturally reduces to pairwise comparisons between the query and the (or a subset of the) structures recorded in the database. The pairwise comparison of RNA secondary structures is a long-standing problem in computational biology, that is still being investigated, as shown by several recent papers, based on different RNA structure representations and computational principles (e.g., [8–12]).

We present here BRASERO, a website that contains several benchmark data sets and automatic software tools to compare the performances of RNA secondary structure comparison methods. The software tools available on BRASERO are flexible and can be used with alternative benchmarks data sets, for example designed by a user with some specific application in mind, with the purpose to assess which models/software tools/parameters are relevant for their own specific application. We describe below the main features of BRASERO and illustrate its use by presenting a short evaluation of several *pairwise comparison* programs based on computing an edit distance or alignment.

## 2. BRASERO Benchmarks and Tools

**Figure 1:**
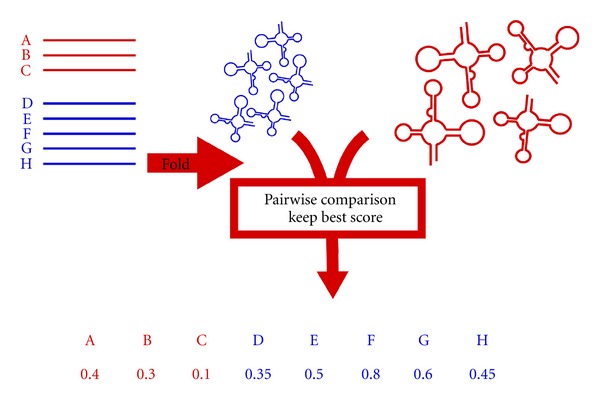
Overview of the BRASERO protocol. The benchmark (left part) is composed of positive (red) and negative (blue) sets of RNA sequences, that are folded and then compared to the reference set (right part). Each comparison tool can be parameterized to specify if it is distance based, in which case lower scores are better, or similarity based, in which case higher scores are better.

A BRASERO benchmark, either provided on BRASERO or designed by a user, aims at assessing the ability of several pairwise RNA secondary structures comparison software tools to properly classify the sequences into positive and negative sets with respect to a given reference set. This assessment is motivated by the practical problem of identifying similar structures (structural homologs) into a large RNA database (see Figure 1).

### 2.1. Structure of a Benchmark

 A benchmark is composed of three sets of RNA (sequences and structures): the *reference*, *positive,* and *negative* sets. For the BRASERO benchmarks currently available, the reference is a set of RNA secondary structures which are all assumed to be members of a same RNA family and for which reliable secondary structures are known; the notion of family or reliable structure could be relaxed in an ad hoc way for specific new benchmarks.

The positive set contains RNA secondary structures that are assumed to belong to the same family as the reference set. The negative set is a set of RNA secondary structures that do not belong to the reference family. More precisely, let *ℱ* be an RNA family. The reference set is denoted by *R*. A set *P*_*s*_ of RNA gene *sequences* that belong to *ℱ* but not to *R* is folded into putative secondary structures using various programs such as mfold [13], RNAshapes [14], or RNAsubopt [15]. For each RNA folding method, both optimal and several suboptimal structures are kept. The set of secondary structures obtained from this folding is the *positive set* and denoted by *P*. Finally, we consider a set *N*_*s*_ of sequences randomly picked from a noise source that is supposed to be free of RNA from *ℱ* and whose lengths have the same distribution as the RNA in *R*. Sequences of *N*_*s*_ are folded (using the same programs and parameters as for *P*_*s*_) to form the *negative set N*.

BRASERO currently comes with data for 5 families: subunit 16S of ribosomal RNAs, microRNAs, small RNAs (sRNAs), Signal Recognition Particles (SRP), and transfer RNAs (tRNAs). The reference genes have been selected manually by the RNA biologists of our team to satisfy the following criteria: accuracy of the structures and inclusion of a large set of possible variations, both in terms of structure and length. To generate sequences of the negative set *F*, we use several sources: viral genomes (from the NCBI Viral Genome Resource) [16], ENCODE sequences [17], and GenRGenS, a generator of random structured sequences [18]. The BRASERO website contains also a documentation on the file formats of a benchmark and the required steps to design a benchmark.

### 2.2. Assessing RNA Comparison Methods Performances

 To assess a pairwise RNA secondary structure comparison method, we compare each structure of *R* with each structure of *T* and *F* using this method. Then for each sequence of *T* and *F* the best score obtained over the comparison of its putative secondary structures and the elements of *R* is kept. Finally, sequences of *T* and *F* are sorted according to these scores. A receiver operating characteristic (ROC) curve is plotted to represent the capability of separating true events (sequences known to be from the *ℱ* family) and false events (sequences not in *ℱ*). This curve shows the false-positive rate *versus* the true-positive rate. The ROC curve of a given benchmark is based on a single run. Indeed, the process of analyzing a benchmark is purely deterministic, the only random aspect lying in the design of the benchmark. For a given RNA family it is possible to design several benchmarks, with several sources (possibly random) of negative sequences.

To perform such experiment with several RNA comparison methods on the same benchmark, a *benchmarking engine* is available on the BRASERO website. It consists of a Java program, that takes as input a benchmark, the considered comparison software tools, and, for every comparison software, a parameters file and a Java class to interface it with the engine. The Java interfaces for several of the classical RNA secondary structure comparison software tools are provided on the website, and a documentation on the format of such interface is also available. For each integrated tool, a Java class indicates if the best score is the smallest (distance approach) or the largest (similarity approach). This information is used to sort the results. Additional Python and Java programs are available to analyze results, to compute ROC curves or to build new benchmarks.

We conclude this section with two important remarks. First the results of a benchmark depend on the method used to fold the positive and negative sets, so our approach can be seen as an evaluation of the combined folding + comparison process. Next, in order to perform a proper assessment of pairwise RNA secondary structure comparison method, the scripts available on the BRASERO website do assume that the RNA structure comparison methods are symmetric and thus do not depend on the order in which two structures are compared. It is up to the users to ensure that the methods they compare satisfy this assumption; classical approach to handle such methods will, for example, average or take the minimum of comparing the structures in both possible orders. Such approaches can easily be implemented in the short JAVA class that has to be written to assess a comparison method (see below).

## 3. Illustration: Comparison Models and the SRP Family

We illustrate here a typical use of the BRASERO website, by comparing several programs based on computing an edit distance or an alignment between pairs of RNA secondary structures, applied on a benchmark for the RNA family of Signal Recognition Particle (SRP). We compare six tools: RNAdistance [19], RNAforester [10], MiGaL [8], TreeMatching [12], Gardenia [9], NestedAlign [20], and RNAStrAT [11]. These tools rely on different models of secondary structures, such as ordered trees, multilayers models, arc-annotated sequences, but are all based on the edit distance and alignment approach pioneered in [19, 21–23]. As these tools also rely on a different usage of the primary sequence conservation, we also included BLAST [24] for comparison. For each software, the default parameters were used.

**Figure 2:**
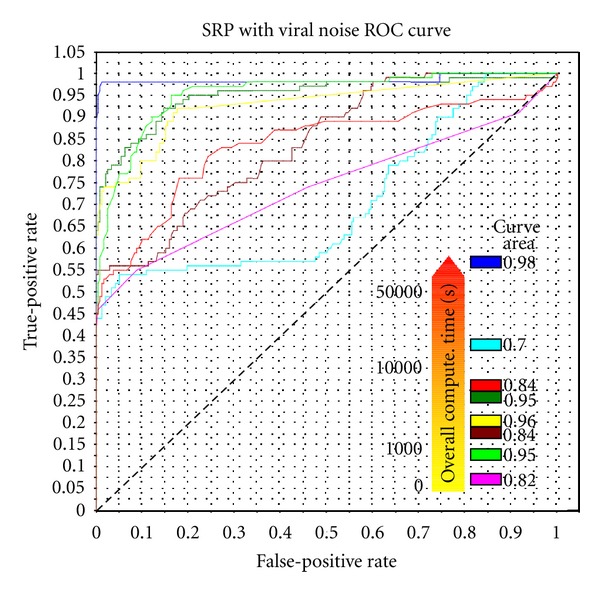
SRP benchmark with 8 pairwise edit distance/alignment methods. ROC curve and computation time. By increasing computation time: BLAST, RNAdistance, Gardenia, NestedAlign, RNAStrAT, Migal, RNAforester, and TreeMatching.

RNAforester is an ordered trees local/global alignment algorithm. It uses a special tree encoding that allows to break nucleotide pairings under certain conditions. MiGaL uses a multilevel representation of the secondary structure composed by four layers coded by rooted ordered trees. The layers model different structural levels from multiloop network to the sequence of nucleotides composing the RNA. The algorithm successively applies edit distance computations to each layer. TreeMatching is based on a quotiented tree representation of the secondary structure which is an autosimilar structure composed of two rooted ordered trees on two different scales (nucleotides and structural elements). The core of the method relies on the comparison of both scales simultaneously: it computes an edit distance between quotiented trees at the macroscopic scale using edit costs defined as edit distances between subtrees at the microscopic scale. Gardenia and NestdAlign use an arc-annotated-based representation, that allows for complex edit operations, such as arc breaking or arc altering. They allow local and global alignment features. Gardenia notably allows affine gap scores, while NestedAlign implements an original local alignment algorithm. RNAStrAT performs the comparison in two steps. First, it compares stems of the two structures using an alignment algorithm with complex edit operations. Then it finds an optimal mapping between the different stems. All tools were used with the default parameters (in particular their default scoring scheme). We applied all tools on a benchmark available on BRASERO for the SRP family benchmark, with noise obtained from viral genomes (details are available on the website). Results are illustrated in Figure 2. Note the choice of the scoring scheme for a given tool may greatly impact the final results and should be evaluated independently before using BRASERO.

We can observe on Figure 2 a clear separation between the software tools based on the principle of computing a global alignment of arc-annotated sequences, and the software tools based on multilayer or hierarchical approaches, that rely on more local alignments. The later seem to perform better, that is, to have a better classification power for the SRP family. Without providing a full analysis of the obtained results, which is beyond the scope of this note, a possible explanation could be that the SRP family exhibits much less sequence and structure conservation than other RNA families (such as tRNA) and that multilayer approaches are able to break down the task of aligning two structures into corresponding sub-structures. This observation, together with its interpretation, can then be used directly in restricting the set of software tools/models to consider when analyzing SRP secondary structures, but also in a longer term perspective by orienting further research specific to this family towards methods based on a multilayer approach.

## 4. Conclusion

BRASERO provides useful tools and benchmarks for comparing RNA secondary structures software tools. Application can be in helping researchers decide on which tool to use either for comparing new RNA secondary structures with a specific family, or in assessing good parameters for pairwise comparison software tools in mining large sets of RNA secondary structures.

Further developments will consist in increasing the number of benchmarks and allowing users to provide their own benchmarks, and in developing additional analysis tools.

## References

[1] Zhu E, Zhao F, Xu G (2010). MirTools: microRNA profiling and discovery based on high-throughput sequencing. *Nucleic Acids Research*.

[2] Sharma CM, Hoffmann S, Darfeuille F (2010). The primary transcriptome of the major human pathogen Helicobacter pylori. *Nature*.

[3] Irnov I, Sharma CM, Vogel J, Winkler WC (2010). Identification of regulatory RNAs in Bacillus subtilis. *Nucleic Acids Research*.

[4] Childs L, Nikoloski Z, May P, Walther D (2009). Identification and classification of ncRNA molecules using graph properties. *Nucleic Acids Research*.

[5] Menzel P, Gorodkin J, Stadler PF (2009). The tedious task of finding homologous noncoding RNA genes. *RNA*.

[6] Gardner PP, Daub J, Tate J (2011). Rfam: wikipedia, clans and the “decimal” release. *Nucleic Acids Research*.

[7] Andronescu M, Bereg V, Hoos HH, Condon A (2008). RNA STRAND: the RNA secondary structure and statistical analysis database. *BMC Bioinformatics*.

[8] Allali J, Sagot MF (2008). A multiple layer model to compare RNA secondary structures. *Software—Practice and Experience*.

[9] Blin G, Denise A, Dulucq S, Herrbach C, Touzet H (2010). Alignments of RNA structures. *IEEE/ACM Transactions on Computational Biology and Bioinformatics*.

[10] Höchsmann M, Töller T, Giegerich R, Kurtz S (2003). Local similarity in RNA secondary structures.. *Proceedings/IEEE Computer Society Bioinformatics Conference*.

[11] Guignon V, Chauve C, Hamel S RNA StrAT: RNA Structure Analysis Toolkit.

[12] Ouangraoua A, Ferraro P, Tichit L, Dulucq S (2007). Local similarity between quotiented ordered trees. *Journal of Discrete Algorithms*.

[13] Markham NR, Zuker M (2005). DINAMelt web server for nucleic acid melting prediction. *Nucleic Acids Research*.

[14] Janssen S, Giegerich R (2010). Faster computation of exact RNA shape probabilities. *Bioinformatics*.

[15] Hofacker IL (2003). Vienna RNA secondary structure server. *Nucleic Acids Research*.

[16] Wheeler DL, Barrett T, Benson DA (2007). Database resources of the National Center for Biotechnology Information. *Nucleic Acids Research*.

[17] Feingold EA, Good PJ, Guyer MS (2004). The ENCODE (ENCyclopedia of DNA Elements) Project. *Science*.

[18] Ponty Y, Termier M, Denise A (2006). GenRGenS: software for generating random genomic sequences and structures. *Bioinformatics*.

[19] Shapiro BA, Zhang K (1990). Comparing multiple RNA secondary structures using tree comparisons. *Computer Applications in the Biosciences*.

[20] Herrbach C (2007). Etude algorithmique et statistique de la comparaison des structures secondaires d’ARN.

[21] Zhang K, Shasha D (1989). Simple fast algorithms for the editing distance between trees and related problems. *SIAM Journal on Computing*.

[22] Jiang T, Wang L, Zhang K (1995). Alignment of trees—an alternative to tree edit. *Theoretical Computer Science*.

[23] Jiang T, Lin G, Ma B, Zhang K (2002). A general edit distance between RNA structures. *Journal of Computational Biology*.

[24] Altschul SF, Gish W, Miller W, Myers EW, Lipman DJ (1990). Basic local alignment search tool. *Journal of Molecular Biology*.

